# A rare case of deafness and renal abnormalities in HDR syndrome caused by a *de novo* mutation in the *GATA3* gene

**DOI:** 10.1590/1678-4685-GMB-2017-0194

**Published:** 2018-11-14

**Authors:** Fábio Tadeu Arrojo Martins, Berenice Dias Ramos, Edi Lúcia Sartorato

**Affiliations:** ^1^ Laboratório de Genética Molecular Humana, Centro de Engenharia Molecular e Genética, Universidade Estadual de Campinas (Unicamp), Campinas, SP, Brazil; ^2^ Departamento de Otorrinolaringologia e Fonoaudiologia Pediátrica, Pontifícia Universidade Católica do Rio Grande do Sul, Porto Alegre, RS, Brazil

**Keywords:** HDR syndrome, hypoparathyroidism, deafness, renal abnormalities, whole exome sequencing

## Abstract

HDR syndrome is a rare autosomal dominant disorder caused by mutations in the *GATA3* gene and characterized by hypoparathyroidism, sensorineural deafness and renal abnormalities. Here we report a Brazilian family, from which the proband, his mother and his grandfather were diagnosed with bilateral sensorineural hearing loss. Molecular screening of the *GJB2*, *GJB6* and *MTRNR1* genes in the proband showed no alterations; however, whole exome sequencing detected a heterozygous mutation, c.1099C > T (p.Arg367*), in the *GATA3* gene. Segregation analyses showed that the mother also had the mutation, but not the grandparents, hence indicating a different hearing impairment type for the grandfather. Paternity test of the mother of the proband confirmed that she has a *de novo* mutation. Furthermore, HDR syndrome was confirmed with new clinical evaluations showing right kidney agenesis in the proband. This is the first study reporting only deafness and renal abnormalities as symptoms of the p.Arg367* mutation in the *GATA3* gene, and also the sixth HDR syndrome case in the world, and the first on the American continent. Together with other reported cases, this study highlights the variability of HDR syndrome symptoms in individuals with the p.Arg367* mutation, emphasizing the importance of molecular analyses for correct diagnosis.

The Barakat Syndrome, also known as HDR syndrome (OMIM 146255), is a very rare disorder associated with an autosomal dominant inheritance pattern and a combination of symptoms, such as hypoparathyroidism, sensorineural deafness and/or renal abnormalities (Barakat *et al.*, 1977; Bilous *et al.*, 1992; Hasegawa *et al.*, 1997; Van Beelen *et al.*, 2014). Among the clinical manifestations, congenital and bilateral sensorineural deafness is the most consistent symptom, ranging from moderate to severe, especially at higher frequencies (Van Beelen *et al.*, 2014). On the other hand, hypoparathyroidism can range from symptomatic or asymptomatic hypocalcemia with low or undetectable parathyroid hormone (PTH) serum levels to paresthesias (Van Esch *et al.*, 2000), while also renal abnormalities present an incomplete penetrance that includes renal dysplasia, hypoplasia, aplasia and vesico-ureteric reflux in one or both kidneys. Importantly, the presence, severity and onset age of the three clinical manifestations can vary among the affected individuals (Van Esch *et al.*, 2000).

Although the reasons for the heterogeneous phenotypes still remain unclear, the HDR syndrome itself has been shown to be caused by alterations that lead to *GATA3* haploinsufficiency (Hasegawa *et al.*, 1997; Van Esch *et al.*, 2000; Muroya *et al.*, 2001; Nesbit *et al.*, 2004; Ferraris *et al.*, 2009; Ohta *et al.*, 2011; Stenson *et al.*, 2017). This gene has six exons and is expressed during embryonic stages (Debacker *et al.*, 1999), generating two isoforms: one with 444 residues (NM_001002295.1) and another with 443 residues (NM_002051.2), of which the latter is considered as the canonical isoform (Uniprot P23771-1). The GATA3 protein is classified as a transcription factor constituted by two transactivating domains (TA1 and TA2) and two highly conserved zinc finger domains: a C-terminal zinc finger (ZnF2), which is essential for DNA binding, and an N-terminal zinc finger (ZnF1) that helps to stabilize the DNA binding, while also interacting with another multi-type zinc finger protein known as Friends of GATA (FOG) (Yang *et al.*, 1994; Nesbit *et al.*, 2004).

We present here the first Brazilian case of HDR syndrome. A European descendant family from the southern region of Brazil presented three individuals diagnosed with hearing loss of unknown etiology (Figure 1A). Of these, the proband (III:2) and his mother (II:2) were diagnosed early in life with bilateral moderate to severe sensorineural hearing loss and both have been using hearing aids since. Furthermore, their hearing loss is non-progressive according to regular audiological evaluations. On the other hand, the maternal grandfather of the proband (I:1) was diagnosed with normal hearing in low frequencies, but with a slope representing severe bilateral hearing loss at 4KHz, after which there is a slight improvement of hearing at 8KHz (Figure 1B). This, together with the fact that the hearing loss of individual I:1 started when he was around 50 years old, exclude the possibility of presbycusis. Moreover, the audiological exams performed at the age of 74 shows a pattern that is typical for noise-induced hearing loss. Audiological tests confirmed normal hearing for the remaining family members.

**Figure 1:**
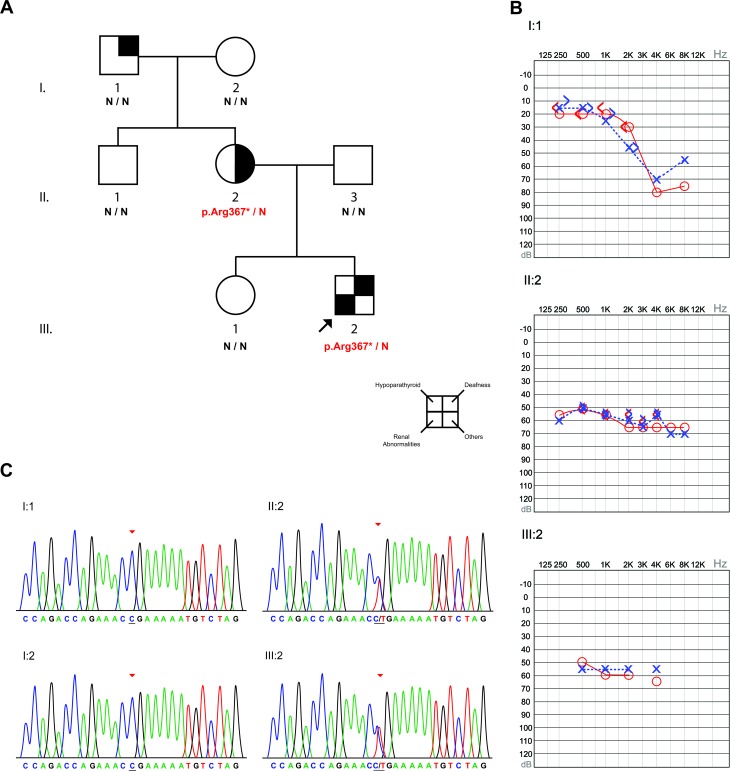
Results of the analyzed family. A) Pedigree of the studied family with the genetic screening for the p.Arg367* mutation in the *GATA3* gene. For the individual II:2, the square denoting other clinical manifestations is marked due to her bicuspid aortic valve. B) Audiograms of the three affected members of the family. Individual I:1 (74 years old) presented normal hearing in low frequencies and a bilateral slope at 4KHz, with an improvement in hearing at 8KHz. The II:2 (37 years old) and III:2 (4 years old) presented bilateral sensorineural moderate to severe hearing loss. C) Electropherograms of the proband (III:2), his mother (II:2) and his grandparents (I:1 and I:2), which indicate a case of *de novo* mutation in the mother of the proband.

Genomic DNA extracted from blood samples of all family members was subjected to primary molecular screening of mutations in the *GJB2* gene, deletions in the *GJB6* gene and the mitochondrial mutation m.A1555G, but no alterations were detected. However, whole exome sequencing (WES) of the proband (III:2) identified a heterozygous c.1099C > T mutation (p.Arg367*) in the *GATA3* gene, which was confirmed by Sanger sequencing. The segregation analysis in the family revealed the presence of this mutation in the mother of the proband (II:2), but not in any of his maternal grandparents, despite the hearing problem of his grandfather (I:1) (Figure 1C). The same molecular results were obtained in repeated tests on a new blood aliquot from the individuals. Nine different human polymorphic markers (*D8S1179*, *D3S1358*, *D5S818*, *D7S820*, *D18S51*, *FGA*, *CSF1PO*, *TH01*, *D21S11*) were genotyped in the I:1, I:2, II:1 and II:2 individuals in order to verify if I:1 and I:2 are the progenitors of II:1 and II:2. This confirmed the paternity of individual II:2 and verified p.Arg367* as a *de novo* mutation in the mother of the proband, as well as the segregation of this mutation to the proband. The mutation frequency was checked using the Brazilian Initiative on Precision Medicine (BIPMed) and the Online Archive of Brazilian Mutations (ABraOM) databases, which both lacked information about the mutation in their 1430 analyzed chromosomes (for details of material and methods see Mat-Met S1 and Table S1 in Supplementary Material). All molecular tests were done in the Human Molecular Genetics laboratory at the Center of Molecular Biology and Genetic Engineering (UNICAMP, Brazil) after all individuals agreed and signed the terms of consent containing all information about the research. The study was approved by the Research Ethics Committee of the Faculty of Medical Sciences of UNICAMP (Process no. 396/2006).

Due to the relationship between the p.Arg367* mutation and the HDR syndrome, an additional investigation regarding hypoparathyroidism and renal abnormalities was conducted and detailed anamneses were remade. The proband presented normal levels of calcium (9.2 mg/dL, normal range: 8.8-11 mg/dL), PTH (12 pg/mL, normal range: 4-58 pg/mL) and phosphate (4.9 mg/dL, normal child range: 4-7 mg/dL). Similarly, the mother (II:2) also presented normal levels for all exams (calcium: 9 mg/dL, normal range: 8.8-11 mg/dL; PTH: 19 pg/mL, normal range: 4-58 pg/mL; phosphate: 4.1 mg/dL, normal adult range: 2.5-5.6 mg/dL). This excluded the presence of hypoparathyroidism in both the proband and his mother.

Regarding renal abnormalities, the proband was diagnosed with right renal agenesis while intrauterine; however, this was never associated to his hearing loss or to a syndrome. Furthermore, the mother has had kidney stones since the age of 33, but neither the proband nor his mother presented proteinuria or hematuria in new blood tests. Nevertheless, it can be concluded that at least the proband has renal abnormalities and, therefore, presents another HDR syndrome clinical manifestation in addition to the hearing loss.

In fact, the HDR syndrome has a very heterogeneous phenotype that varies even within a family with the same mutation. Ferraris *et al.* (2009) studied the HDR clinical manifestations in 77 patients, and found that 62.3% presented hypoparathyroidism, deafness and renal abnormalities, while 28.6% presented only hypoparathyroidism and deafness. Deafness coupled with renal abnormalities constituted only 2.6% of the cases, which is the case for the family in our study.

The p.Arg367* mutation identified in the proband in this study results in a premature stop codon, thereby shortening the GATA3 protein by 77 amino acids. The mutation occurs after the ZnF2 domain, but is predicted to delete the C-terminal part of the protein, which is essential for DNA binding (Omichinski *et al.*, 1993; Van Esch *et al.*, 2000; Nesbit *et al.*, 2004). The mutation was first reported in an individual from a Japanese family (Muroya *et al.*, 2001). The variant was not present in the parents or the sister, indicating a *de novo* mutation in the proband. All three symptoms were present in the proband, while her daughter inherited the mutation, but with sensorineural deafness as the only symptom. Another study found the same mutation in a family from Northern Europe, of which one individual presented hypoparathyroidism and deafness without any renal problems. None of the parents had the mutation and, hence, this was a case of the same *de novo* mutation (Nesbit *et al.*, 2004). Furthermore, two children in a Chinese family were clinically diagnosed with HDR syndrome and both were heterozygous for the p.Arg367* mutation with hypoparathyroidism and deafness as symptoms. Again, none of the parents had the mutation and the paternity was further confirmed by tests using four polymorphic markers, which lead to a final hypothesis that the mutation was caused by germinal mosaicism (Sun *et al.*, 2009). The latest report of HDR syndrome caused by the p.Arg367* mutation in the *GATA3* gene is a male infant from a Turkish family, who presented hypoparathyroidism and renal anomalies, while auditory tests were normal. This was also classified as a *de novo* mutation, since it was not found in the parents of the proband (Döneray *et al.*, 2015) (Table 1).

**Table 1:** All reported clinical results involving the p.Arg367* mutation associated with HDR syndrome.

Gender	*de novo* mutation	Hypoparathyroidism (H)				Sensorineural deafness (D)		Renal abnormalities (R)		Ethnic group	Reference
		Diagnosis (onset age)	Calcium	Phosphate	PTH	Diagnosis (onset age)	Phenotype	Diagnosis (onset age)	Phenotype		
			(mg/dL)	(mg/dL)	(pg/mL)						
M	-	-	9.2	4.9	12	11 months*	Bilateral (Moderate/Severe)	Intrauterine	Right renal agenesis	Brazilian	*This study*
								(28 weeks)			
F	+	-	9	4.1	19	4 years*	Bilateral (Moderate/Severe)	-	Kidney stones	Brazilian	*This study*
M	+	2 months	6.4	7.2	7	-	-	2 months	Mild proteinuria and hematuria	Turkish	(Döneray *et al*., 2015)
M	+	10 years	6.5	9.1	1.6	3 years	Bilateral	10 years	Mild proteinuria	Chinese	(Sun *et al*., 2009)
F	+	5 years	8	-	3	5 years	Not informed	-	-	Chinese	(Sun *et al*., 2009)
M	+	13 years	4.2**	-	6	Less than 1 year	Bilateral (R [+GT+] L)	-	-	Northern European	(Nesbit *et al*., 2004)
F	+	3 years	8.1**	3.5**	Undetected	Probably* (childhood)	Not examined	Probably in the 2^nd^ decade	Proteinuria and hematuria	Japanese	(Muroya *et al*., 2001)

M: Male; F: Female

* With Hearing aids

** Converted values from mmol/L to mg/dL

To our knowledge, there are no studies regarding the frequency of HDR syndrome in the world population, but it can be considered as a rare disorder (Van Beelen *et al.*, 2014). Regarding the HDR syndrome caused by the p.Arg367* mutation, our study represents the sixth reported case and the first one on the American continent. Our analyses concluded that the hearing loss in the studied family was not non-syndromic as first suspected, but rather the result of the HDR syndrome. These findings are supported by the occurrence of the p.Arg367* mutation in the *GATA3* gene in the proband and his mother. Importantly, the proband in our study was diagnosed with deafness and renal abnormalities, which is the first report of these two symptoms as the only clinical manifestations caused by the p.Arg367* mutation, thereby confirming the high phenotype heterogeneity associated with this syndrome. Despite the same genotype, the symptoms vary between individual II:2 with only sensorineural hearing loss and individual III:2 with both hearing loss and renal abnormalities, which is well in accordance with the heterogeneity aspect. Furthermore, this study, together with the studies of the Turkish and Northern Europe families, contradicts the belief that the p.Arg367* mutation is exclusively found in Far East populations (Nesbit *et al.*, 2004; Döneray *et al.*, 2015;).

The results presented here also show how molecular diagnostic can aid the correct clinical diagnosis of affected individuals. Proper and complete diagnosis of a condition, coupled to efficient genetic counseling, can prevent an infant from developing more severe symptoms in adult life, since the chances of correct treatment and the ability to anticipate medical problems are markedly increased. Hence, a decrease of patient life quality can be counteracted and possibly even prevented. In the case of HDR syndrome, the clinical heterogeneity makes screening of the *GATA3* gene both important and worthwhile in suspected cases.

## Supplementary material

The following online material is available for this article:

Mat-Met S1 - Description of all material and methods used in this study.

Table S1 - Detailed list of primers used in the study.
