# Supplementary material for: A rare case of deafness and renal abnormalities in HDR syndrome caused by a de novo mutation in the GATA3 gene
Source: Genet Mol Biol. 2018 Nov 14;41(4):794–8. doi: 10.1590/1678-4685-GMB-2017-0194 (PMC6415598; doi:10.1590/1678-4685-GMB-2017-0194)
Supplement: Supplementary file 1 [file 1415-4757-GMB-1678-4685-GMB-2017-0194-s001.pdf]

Supplementary Material to "A rare case of deafness and renal abnormalities in HDR syndrome caused by a *de novo* mutation in the *GATA3* gene"

Mat Met S1 - Detailed Material and Methods

Casuistics

Three individuals from a three generation Brazilian family that had previously been diagnosed with bilateral sensorineural hearing loss were submitted to primary molecular screening for genetic causes for the hearing loss. Five milliliters of peripheral blood were collected from each individual in a Lavender Vacutainer®, after which genomic DNA was extracted from blood leukocytes by standard phenol-chloroform extraction. The concentration and purity were checked by Qubit® dsDNA BR Assay Kit with Qubit 2.0 (Life Technologies, CA, USA) and NanoDrop (Thermo Scientific, MA, USA), respectively.

Primary molecular screening for hearing loss

In the primary molecular screening, the coding and flanking regions of the *GJB2* gene were sequenced, and the *GJB6* gene was analyzed for the two main deletions *GJB6-D13S1830* and *GJB6-D13S1854* analyzed by Multiplex PCR (F. del Castillo *et al*., 2005; I. Del Castillo *et al*., 2003). Furthermore, the m.A1555G mutation in the *MT-RNR1* gene was analyzed by RFLP-PCR and *BsmAI* endonuclease according to manufacturer recommendations (New England Biolabs, MA, USA). Table S1 lists all primers used in the study.

Standard PCR

The standard PCR reaction was optimized using 0.3 μM of each primer, 50 ng of template DNA, 1.5 mM of $MgCl_2$, 200 μM of dNTP, 1X reaction buffer (50 mM KCl, 20 mM Tris-HCl, pH 8.4) and 1 U *Taq* polymerase. The final volume was completed to 25 μL with ultrapure water. A standard PCR cycling program was used for all reactions, with the Tm varying according to the primers used (Table S1). The PCR products were analyzed on a 1% agarose gel dyed with

Ethidium Bromide and then purified using the Wizard SV gel and PCR clean-up system (Promega, WI, USA).

Sanger sequencing

The samples were prepared using BigDye Terminator v3.1 Cycle Sequencing (Thermo Scientific, MA, USA) and the EDTA-ethanol purification protocol, and subsequently sequenced using a 3730xL sequencer (Applied Biosystems, CA, USA) from LaCTAD/UNICAMP (Brazil). The sequence was aligned to the wild type sequence and the peaks were analyzed with the Benchling tool (Benchling Inc., CA, USA).

Whole exome sequencing and bioinformatics analyses

The paired-end (2 x 100 bp) exonic library was prepared using the Nextera Expanded Exome Kit (Illumina, CA, USA) according to manufacturer recommendations. The HiSeq2500 sequencer (Illumina, CA, USA) at LaCTAD/UNICAMP was used for sample sequencing. The resulting raw data was aligned by Burrows-Wheeler Aligner (BWA) using the hg38 genome version. The data were processed with FastQC (Babraham Institute, UK), Trimmomatic (Bolger *et al*., 2014), Picard tools (Broad Institute Inc., USA), SamTools (Li *et al*., 2009), and GATK 3.7 tools (Van Der Auwera *et al*., 2014; DePristo *et al*., 2011). The Variant Call Format (vcf) file was annotated by wANNOVAR (Chang *et al*., 2012) and the variants were analyzed by customized filters. The candidate variant was confirmed by Sanger sequencing, along with in the segregation analysis of the family.

Frequency analysis

The Brazilian Initiative on Precision Medicine (BIPMed, 2017) and the Online Archive of Brazilian Mutations (ABraOM) databases (Naslavsky *et al*., 2017) were used for frequency analysis of the p.Arg367* mutation in the Brazilian population.

Paternity tests

Nine polymorphic markers (*D3S1358*, *D5S818*, *D7S820*, *D8S1179*, *D18S51*, *D21S11*, *CSF1PO*, *TH01* and *FGA*) were selected from the CODIS (COmbined DNA Index System) database (Federal Bureau of Investigation, USA) to confirm the paternity in the studied family and, hence, the familial case of *de novo* mutation. All markers were amplified by following the standard PCR conditions. The amplicons were checked using a 5% UltraPure™ agarose-1000 gel (Thermo Scientific, MA, USA). The fragments were dyed with Ethidium Bromide and cut and purified using the Wizard SV gel and PCR clean-up system (Promega, WI, USA). The polymorphic markers were analyzed by Sanger sequencing using the primers in Table S1.

References

Van Der Auwera GA, Carneiro MO, Hartl C, Poplin R, Levy-moonshine A, Jordan T, Shakir K, Roazen D, Thibault J, Banks E, *et al* (2014) From FastQ data to high confidence varant calls: the Genonme Analysis Toolkit best practices pipeline. Current Protocols in Bioinformatics 11: 1-33.

BIPMed. The Brazilian Initiative on Precision Medicine. (2017). Retrieved from http://bipmed.iqm.unicamp.br

Bolger AM, Lohse M and Usadel B (2014). Trimmomatic: A flexible trimmer for Illumina sequence data. Bioinformatics, 30: 2114–2120.

Chang X and Wang K (2012) wANNOVAR: annotating genetic variants for personal genomes via the web. Journal of Medical Genetics, 49: 433–436.

del Castillo F, Rodríguez-Ballesteros M, Alvarez A, Hutchin T, Leonardi E, de Oliveira C, Azaiez H, Brownstein Z, Avenarius MR, Marlin S, *et al*. (2005). A novel deletion involving the connexin-30 gene, del(GJB6-d13s1854), found in trans with mutations in the GJB2 gene (connexin-26) in subjects with DFNB1 non-syndromic hearing impairment. Journal of Medical Genetics, 42:588–594.

Del Castillo I, Moreno-Pelayo MA, Del Castillo FJ, Brownstein Z, Marlin S, Adina Q, Cockburn DJ, Pandya A, Siemering KR, Chamberlin GP, *et al.* (2003). Prevalence and evolutionary

origins of the del(GJB6-D13S1830) mutation in the DFNB1 locus in hearing-impaired subjects: a multicenter study. Am J Hum Genet, 73:1452–1458.

DePristo MA, Banks E, Poplin RE, Garimella KV, Maguire JR, Hartl C, Philippakis AA, del Angel G, Rivas MA, Hanna M, *et al*. (2011). A framework for variation discovery and genotyping using next- generation DNA sequencing data. Nature Genetics, 43:491–498.

Li H, Handsaker B, Wysoker A, Fennell T, Ruan J, Homer N, Marth G, Abecasis G, Durbin R (2009). The Sequence Alignment/Map format and SAMtools. Bioinformatics, 25:2078–2079.

Naslavsky MS, Yamamoto GL, de Almeida TF, Ezquina SAM, Sunaga DY, Pho N, Bozoklian D, Sandberg TOM, Brito LA, Lazar M, *et al*. (2017). Exomic variants of an elderly cohort of Brazilians in the ABraOM database. Human Mutation, 38:1–13.
