# Supplementary material for: A rare case of deafness and renal abnormalities in HDR syndrome caused by a de novo mutation in the GATA3 gene
Source: Genet Mol Biol. 2018 Nov 14;41(4):794–8. doi: 10.1590/1678-4685-GMB-2017-0194 (PMC6415598; doi:10.1590/1678-4685-GMB-2017-0194)
Supplement: Supplementary file 2 [file 1415-4757-GMB-1678-4685-GMB-2017-0194-s002.pdf]

Supplementary Material to "A rare case of deafness and renal abnormalities in HDR syndrome caused by a *de novo* mutation in the *GATA3* gene"

Table S1 - Detailed list of primers used in the study.

Gene	Reaction	Primer Forward (*5'>3'*)	Primer Reverse (*5'>3'*)	Tm (ºC)	Fragment size (bp)
GJB2	Coding region	CTCCCTGTTCTGTCCTAGC	GGTTGCCTCATCCCTC	54	815
GJB6	GJB6-	TCATAGTGAAGAACTCGATGCTGTTT	CAGCGGCTACCCTAGTTGTGGT	62	460
	GJB6-	CACCATGCGTAGCCTTAACCATTTT	TTTAGGGCATGATTGGGGTGATTT	62	564
	Control	CGTCTTTGGGGGTGTTGCTT	CATGAAGAGGGCGTACAAGTTAGA	62	333
MT-RNR1	m.A1555G	ATATCTGAACACACAATAGC	GAAACCGACCTGGATTACTC	54	2060
GATA3	Exon 6	GAGACAGGAGAAGGTGGGAG	CTCTAACCCATGGCGGTGA	59	446
STRs	*D8S1179*	CTTGGGGTGTCGCTTTTCT	TGCATTGTTGTTGGGAATGT	60	396
	D3S1358	AGCTATTCCCAGGTGAGGAC	TGAGATGGGAGGATCACTTG	58	466
	D5S818	CCCATCTGGATAGTGGACCT	TTTTAAAATTAAATACTGAGACATGC	58	374
	D7S820	CGATTCCACATTTATCCTCA	AGTGCAGTGGTGCGATCT	57	393
	D18S51	CAAAAATTAGTTGGGCATGG	CCGACTACCAGCAACAACAC	58	400
	FGA	GCAGAAGCTGGATATGCTGT	CATCTTAACTGGCATTCATGG	58	393
	CSF1PO	TCAGACTCTTCCACACACCA	GAGTGATGGGGTCAGATTGT	57	400
	TH01	CTCCTGTGGGCTGAAAAG	TAGTCAGCACCCCAACCA	55	299
	D21S11	AGACTTGGACAGCCACACTG	AAACACTGAGAAGGGAGAAACA	58	400
